# Supplementary material for: A pan-variant miniprotein inhibitor protects against SARS-CoV-2 variants
Source: bioRxiv. 2024 Aug 10:2024.08.08.606885. Preprint. [Version 1] doi: 10.1101/2024.08.08.606885 (PMC11326246; doi:10.1101/2024.08.08.606885)
Supplement: 1 [file NIHPP2024.08.08.606885v1-supplement-1.pdf]

bioRxiv preprint doi: https://doi.org/10.1101/2024.08.08.606885; this version posted August 10, 2024. The copyright holder for this preprint (which was not certified by peer review) is the author/funder, who has granted bioRxiv a license to display the preprint in perpetuity. It is made available under aCC-BY 4.0 International license.

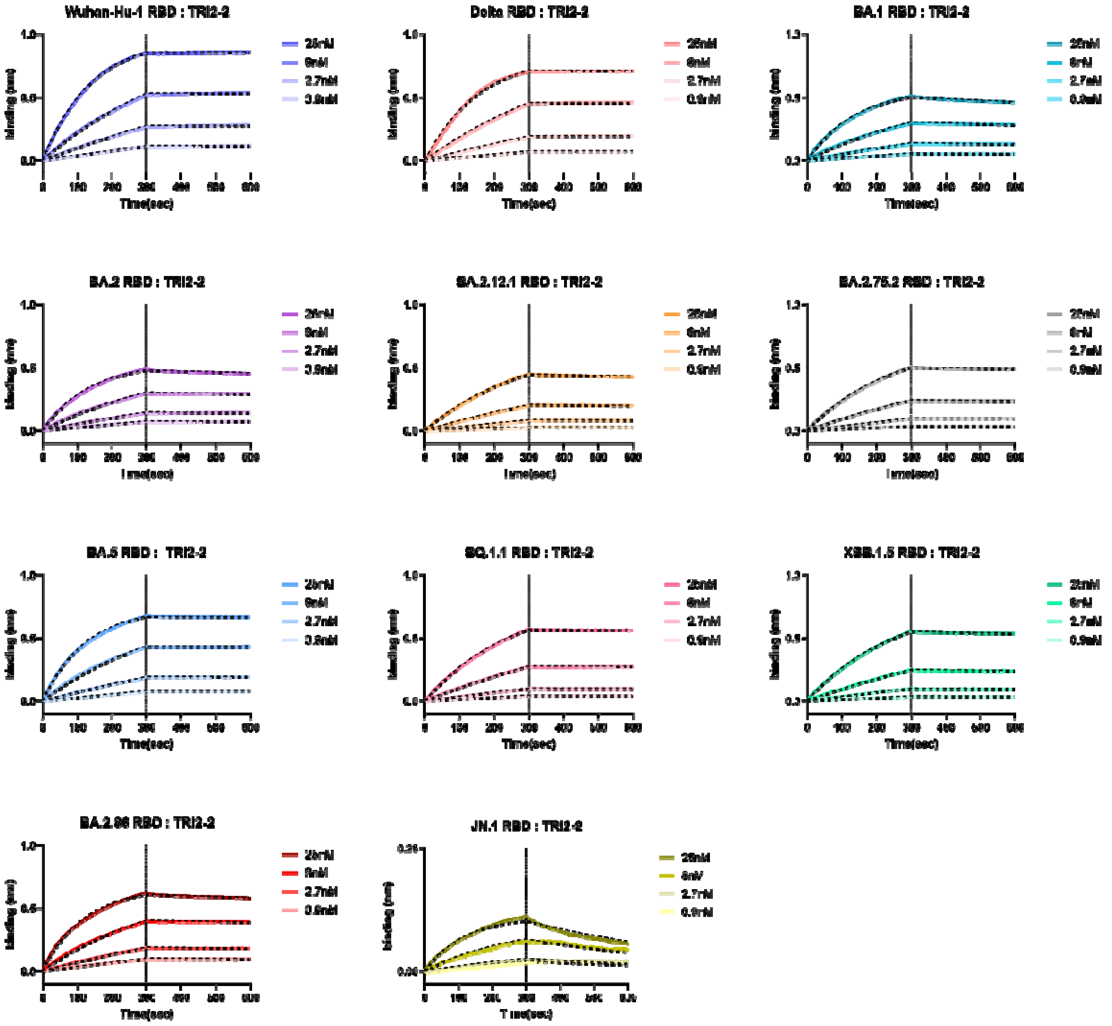

Supplementary Figure 1. Kinetic analysis of TRI2-2 binding to SARS-CoV-2 variant RBDs immobilized on using biolayer interferometry. Biotinylated RBDs were immobilized on streptavidin biosensors to a final level of 1 nm shift each. The TRI2-2 concentrations used are provided in the color keys. Dashed black lines represent curve fits obtained using global fitting and a 1:1 binding model in the ForteBio BLI software. Representative graphs are shown from two biological replicates.

bioRxiv preprint doi: https://doi.org/10.1101/2024.08.08.606885; this version posted August 10, 2024. The copyright holder for this preprint (which was not certified by peer review) is the author/funder, who has granted bioRxiv a license to display the preprint in perpetuity. It is made available under aCC-BY 4.0 International license.

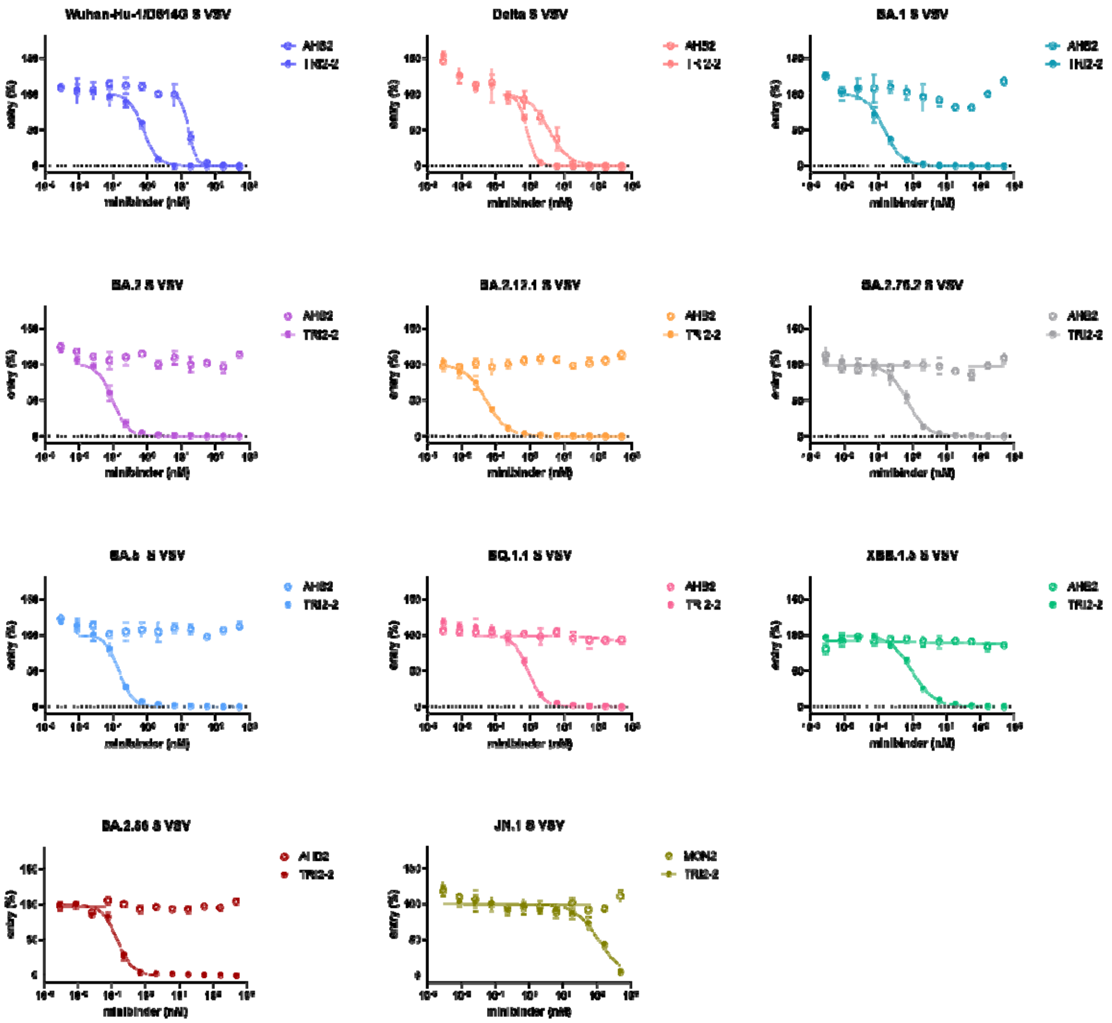

Supplementary Figure 2. Dose-response curves for neutralization of SARS-CoV-2 S variant VSV pseudoviruses by the TRI2-2 and AHB2 miniprotein inhibitors. Each dot represents the mean of three technical replicates. SD shown as lines. Representative graphs are shown from three biological replicates.

bioRxiv preprint doi: https://doi.org/10.1101/2024.08.08.606885; this version posted August 10, 2024. The copyright holder for this preprint (which was not certified by peer review) is the author/funder, who has granted bioRxiv a license to display the preprint in perpetuity. It is made available under aCC-BY 4.0 International license.

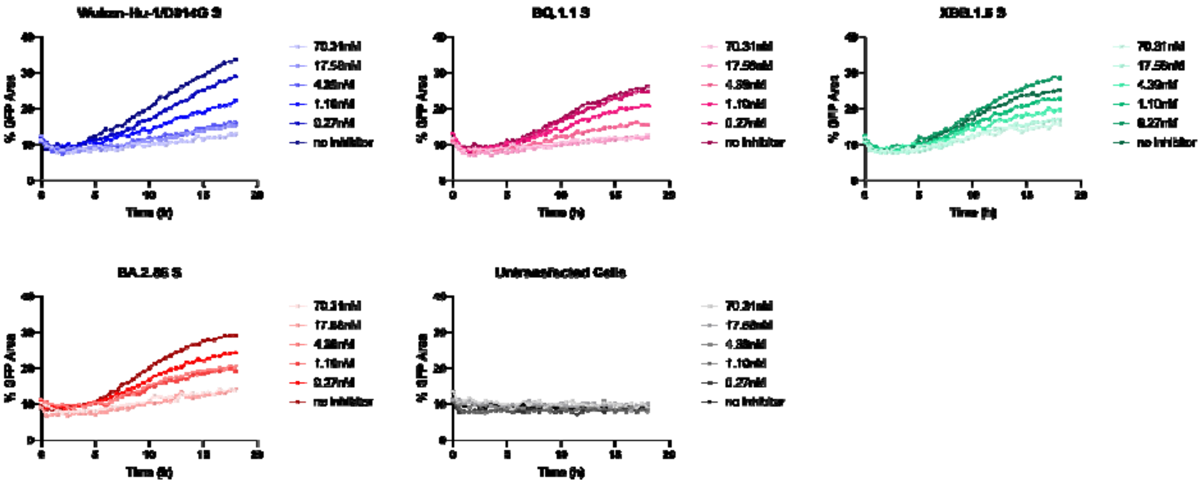

Supplementary Figure 3. Dose-response curves for TRI2-2-mediated fusion inhibition of SARS-CoV-2 S variants. Representative graphs are shown from four biological replicates.

bioRxiv preprint doi: https://doi.org/10.1101/2024.08.08.606885; this version posted August 10, 2024. The copyright holder for this preprint (which was not certified by peer review) is the author/funder, who has granted bioRxiv a license to display the preprint in perpetuity. It is made available under aCC-BY 4.0 International license.

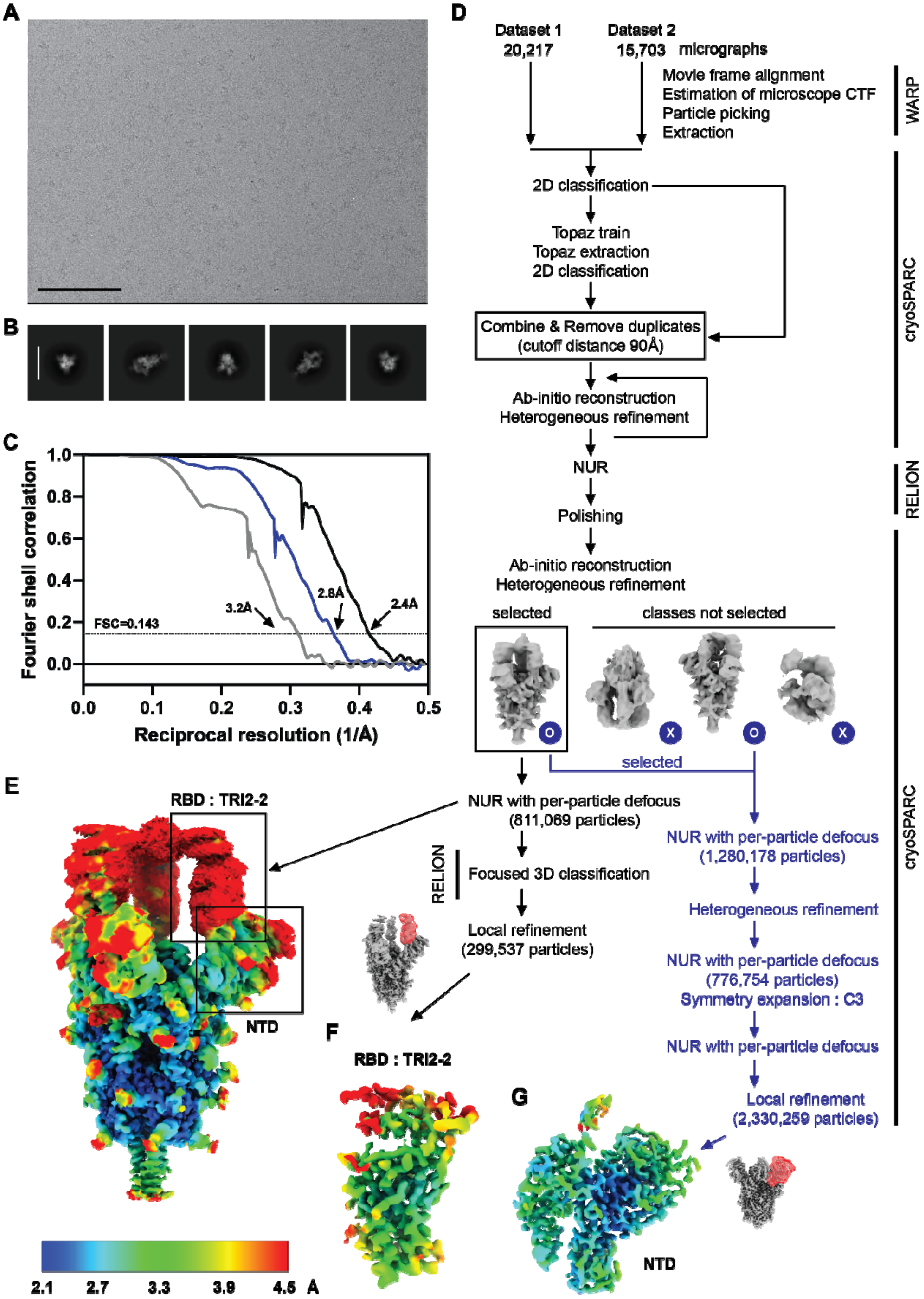

bioRxiv preprint doi: https://doi.org/10.1101/2024.08.08.606885; this version posted August 10, 2024. The copyright holder for this preprint (which was not certified by peer review) is the author/funder, who has granted bioRxiv a license to display the preprint in perpetuity. It is made available under aCC-BY 4.0 International license.

Supplementary Figure 4. CryoEM data collection and refinement of TRI2-2 bound to the BA.2.86 S glycoprotein trimer. (A-B) Representative electron micrograph (A) and 2D class averages (B) of SARS-CoV-2 BA.2.86 S in complex with TRI2-2. The scale bar represents 100nm (A) and 210Å (B). **(C)** Gold-standard Fourier shell correlation curves for the cryoEM reconstructions. The 0.143 cutoff is indicated with a gray dashed line. Black, gray, and blue curves correspond to the global, RBD, and NTD reconstructions, respectively. **(D)** Data processing flowchart. NUR: non-uniform refinement. Masks used for local refinement are shown in red. **(E-G)** CryoEM map of SARS-CoV-2 BA.2.86 S in complex with TRI2-2 (E), locally refined map of the BA.2.86 RBD in complex with TRI2-2 (F), and locally refined map of the BA.2.86 NTD (G) colored by local resolution as determined using cryoSPARC.

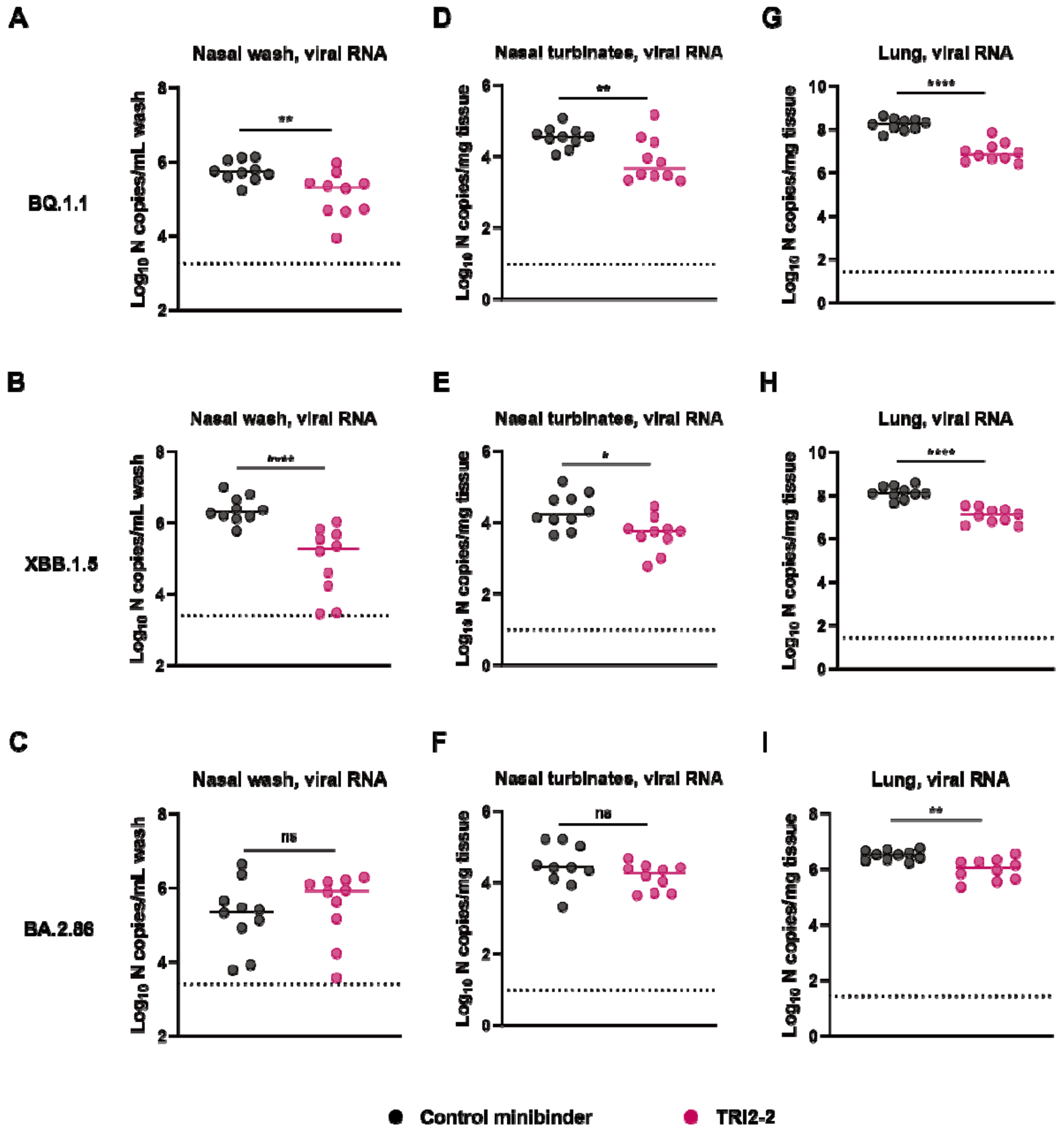

Supplementary Figure 5. Quantification of viral RNA loads. (A-C) Genomic viral RNA levels in nasal washes for mice challenged with BQ.1.1 (A), XBB.1.5 (B), or BA.2.86 (C). **(D-F)** Genomic viral RNA levels in nasal turbinates for mice challenged with BQ.1.1 (D), XBB.1.5 (E), or BA.2.86 (F). **(G-I)** Genomic viral RNA levels in lungs for mice

bioRxiv preprint doi: https://doi.org/10.1101/2024.08.08.606885; this version posted August 10, 2024. The copyright holder for this preprint (which was not certified by peer review) is the author/funder, who has granted bioRxiv a license to display the preprint in perpetuity. It is made available under aCC-BY 4.0 International license.

challenged with BQ.1.1 (G), or XBB.1.5 (H), or BA.2.86 (I) (lines indicate median; n = 10 mice per group per virus challenge, two experiments; Two-tailed Mann-Whitney test between control and TRI2-2 treatment; ns, not significant; *P < 0.05, **P<0.01, ***P < 0.001, ****P < 0.0001.).

Supplementary Table 1. Representative TRI2-2 binding kinetics and avidities (apparent affinities denoted $K_{D,app}$) to SARS-CoV-2 variant RBDs obtained by biolayer interferometry. Values shown here are calculated from the curve fit from Supplementary Figure 1.

	$K_{D,app}$ (M)	$K_{D,app}$ error	k_{on} (1/Ms)	k_{on} error	k_{off} (1/s)	k_{off} error
**D614G**	1.21E-12	N/A	2.86E+05	2.42E+02	3.46E-07	N/A
**Delta**	1.14E-12	N/A	2.59E+05	4.17E+02	2.95E-07	N/A
**BA.1**	9.28E-10	6.07E-12	2.84E+05	5.09E+02	2.64E-04	1.66E-06
**BA.2**	4.93E-10	5.82E-12	2.97E+05	5.47E+02	1.47E-04	1.71E-06
**BA.2.12.1**	8.51E-10	8.90E-12	1.37E+05	2.93E+02	1.17E-04	1.20E-06
**BA.2.75.2**	5.42E-10	6.40E-12	1.65E+05	2.67E+02	8.93E-05	1.05E-06
**BA.5**	3.06E-11	4.60E-12	3.25E+05	4.97E+02	9.92E-06	1.49E-06
**BQ.1.1**	2.10E-10	5.49E-12	1.60E+05	2.23E+02	3.36E-05	8.78E-07
**XBB.1.5**	6.58E-10	7.02E-12	1.60E+05	2.78E+02	1.05E-04	1.11E-06
**BA.2.86**	3.88E-10	5.79E-12	3.39E+05	6.69E+02	1.31E-04	1.95E-06
**JN.1**	6.19E-09	6.11E-11	2.82E+05	2.42E+03	1.75E-03	8.53E-06

Supplementary Table 2. CryoEM data collection and refinement statistics.

	**SARS-CoV-2 S BA.2.86 in complex with TRI2-2 minibinder**	**SARS-CoV-2 BA.2.86 RBD in complex with TRI2-2 minibinder**	**SARS-CoV-2 BA.2.86 NTD**
**Data collection and processing**	EMD-45972<br>PDB 9CWR	EMD-45969<br>PDB 9CWP	EMD-45971<br>PDB 9CWQ

bioRxiv preprint doi: https://doi.org/10.1101/2024.08.08.606885; this version posted August 10, 2024. The copyright holder for this preprint (which was not certified by peer review) is the author/funder, who has granted bioRxiv a license to display the preprint in perpetuity. It is made available under aCC-BY 4.0 International license.

**Magnification**	**105,000**	**105,000**	**105,000**
**Voltage (kV)**	**300**	**300**	**300**
**Electron exposure ($e^{-}/Å^{2}$)**	**53.25**	**53.25**	**53.25**
**Defocus range (μm)**	**-0.8 - -1.8**	**-0.8 - -1.8**	**-0.8 - -1.8**
**Pixel size (Å)**	**0.829**	**0.829**	**0.829**
**Symmetry imposed**	**C1**	**C1**	**C1**
**Final particle images (no.)**	**811,069**	**299,537**	**2,330,259**
**Map resolution (Å)**	**2.4**	**3.2**	**2.8**
**FSC threshold**	**0.143**	**0.143**	**0.143**
**Map sharpening B factor ($Å^{2}$)**	**-76.2**	**-117.8**	**-91.7**
**Validation**			
**MolProbity score**	**1.02**	**0.82**	**1.64**
**Clashscore**	**1.80**	**0.79**	**2.33**
**Poor rotamers (%)**	**0.95**	**0.54**	**2.69**
**Ramachandran plot**			
**Favored (%)**	**97.63**	**97.68**	**95.73**
**Allowed (%)**	**2.34**	**2.32**	**4.27**
**Disallowed (%)**	**0.03**	**0.00**	**0.00**
